# Interactive effects of climate, land use and soil type on *Culex pipiens/torrentium* abundance

**DOI:** 10.1016/j.onehlt.2023.100589

**Published:** 2023-06-21

**Authors:** Louie Krol, Rody Blom, Martha Dellar, Jordy G. van der Beek, Arjan C.J. Stroo, Peter M. van Bodegom, Gertjan W. Geerling, Constantianus J.M. Koenraadt, Maarten Schrama

**Affiliations:** a Institute of Environmental Sciences, Leiden University, the Netherlands; b Deltares, Daltonlaan 600, Utrecht, the Netherlands; c Laboratory of Entomology, Wageningen University and Research, Wageningen, the Netherlands; d Centre for Monitoring of Vectors, Netherlands Food and Consumer Product Safety Authority, Ministry of Agriculture, Nature and Food Quality, Wageningen, the Netherlands; e Department of Environmental Science, Radboud Institute for Biological and Environmental Sciences, Radboud University, Nijmegen, the Netherlands

**Keywords:** Mosquito-borne diseases, One health, Water management, Mosquito surveillance, West Nile virus, Usutu virus

## Abstract

The incidence and risk of mosquito-borne disease outbreaks in Northwestern Europe has increased over the last few decades. Understanding the underlying environmental drivers of mosquito population dynamics helps to adequately assess mosquito-borne disease risk. While previous studies have focussed primarily on the effects of climatic conditions (i.e., temperature and precipitation) and/or local environmental conditions individually, it remains unclear how climatic conditions interact with local environmental factors such as land use and soil type, and how these subsequently affect mosquito abundance.

Here, we set out to study the interactive effects of land use, soil type and climatic conditions on the abundance of *Culex pipiens/torrentium*, highly abundant vectors of West Nile virus and Usutu virus. Mosquitoes were sampled at 14 sites throughout the Netherlands. At each site, weekly mosquito collections were carried out between early July and mid-October 2020 and 2021. To assess the effect of the aforementioned environmental factors, we performed a series of generalized linear mixed models and non-parametric statistical tests.

Our results show that mosquito abundance and species richness consistently differ among land use- and soil types, with peri-urban areas with peat/clay soils having the highest *Cx. pipiens/torrentium* abundance and sandy rural areas having the lowest. Furthermore, we observed differences in precipitation-mediated effects on *Cx. pipiens/torrentium* abundance between (peri-)urban and other land uses and soil types. In contrast, effects of temperature on *Cx. pipiens/torrentium* abundance remain similar between different land use and soil types.

Our study highlights the importance of both land use and soil type in conjunction with climatic conditions for understanding mosquito abundances. Particularly in relation to rainfall events, land use and soil type has a marked effect on mosquito abundance. These findings underscore the importance of local environmental parameters for studies focusing on predicting or mitigating disease risk.

## Graphical abstract

**Figure:**
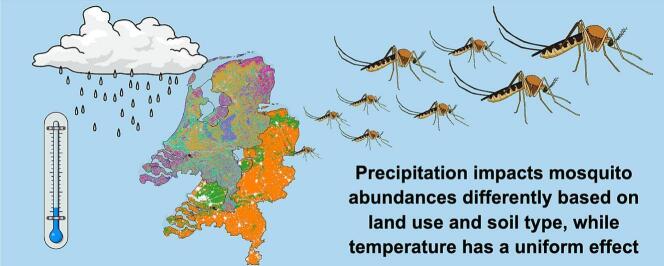

## 1 Introduction

In recent years, we have witnessed an increase in mosquito-borne disease outbreaks, both in the Netherlands and in Europe at large; these have included Usutu virus (USUV) and West Nile virus (WNV), both of which are predominantly transmitted by *Culex pipiens* mosquitoes [[1], [2], [3], [4], [5], [6], [7]]. Mosquito-borne disease outbreaks and future risk are often studied from the perspective of changes in temperature and precipitation resulting from global climate change [[8], [9], [10]]. However, the heterogeneity in mosquito abundance and disease incidence that exists at the landscape scale illustrates that in addition to climate, other factors are likely to play an important role [[11], [12], [13], [14], [15], [16], [17], [18], [19]]. Results of recent modeling and field studies suggest that factors related to land use and soil type may play a significant role in driving mosquito abundance and community composition [[20], [21], [22], [23], [24], [25]]. Land use patterns are thought to influence mosquito abundance due to differences in the availability of aquatic habitats, food availability, predation, host availability, vegetation and capacity to buffer temperature effects [[23], [24], [25], [26], [27], [28], [29], [30]]. Soil type affects local hydrology, related to the persistence of aquatic habitat, and water chemistry, which is linked to food availability [[30], [31], [32], [33]]. Unlike for soil type, no comprehensive GIS layers exist for these more direct predictors of mosquito abundances. These factors related to land use and soil type have been shown to be important mosquito population regulation factors [32,33]. However, despite inclusion of soil type and land use in some models, it remains poorly understood how and to what extent differences in soil types and land uses play a role in driving mosquito abundance, and how these interact with temperature and precipitation [27,34]. Here, we aim to explore the independent and interactive effects of land use, soil type, temperature and precipitation. To this end, we conducted a field study at fourteen research sites, over the course of two consecutive summers (2020 and 2021). In this study, we focus largely on *Cx. pipiens/torrentium*, two of the most widespread and abundant mosquito species in Northwest Europe which have a wide tolerance for temperature and eutrophication levels and are known to occupy a wide variety of aquatic habitats [2,35].

## 2 Methods

### 2.1 Mosquito sampling

**Fig. 1:**
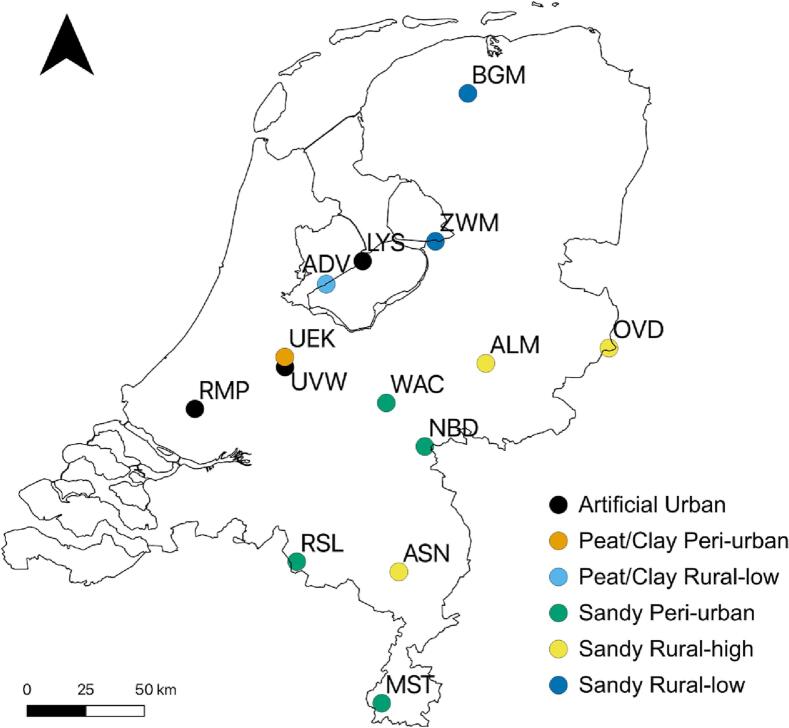
Maps depicting the 14 mosquito sampling sites in the Netherlands grouped into six soil type/land use classes: peat/clay rural low (ADV), sandy rural high (ALM, ASN, OVD), sandy rural low (BGM, ZWM), artificial urban (LYS, RMP, UVW), sandy peri-urban (MST, NBD, RSL, WAC), and peat/clay peri-urban (UEK).

Mosquitoes were collected at 14 established bird ringing sites distributed across a range of soil types and land uses in the Netherlands (Fig. 1). At each sampling site, mosquitoes were collected on a weekly basis for one night per week by volunteers from early July until mid-October in 2020 and 2021 (fig. S1). Mosquitoes were trapped with CO_2_-baited BG-Pro traps (Biogents GmbH, Regensburg, Germany) without additional lures. CO_2_ was produced following a slightly modified molasses fermentation protocol [36]. A mixture of 250 g sugarcane molasses (Agriton), 17.5 g dried instant yeast (Bruggeman instant yeast, Algist Bruggeman, Belgium) and ∼ 1.7 l of tap water was added to 5-l jerrycans. The jerrycans were connected to the mosquito traps via silicone tubes (Ø7 mm; RubberBV, Hilversum, the Netherlands). Mosquito traps were placed 1–1.5 m above ground level. The collected female mosquitoes were identified morphologically using the identification characteristics from Becker et al., (2020) [35]. *Culex pipiens s.l.* and its sibling species *Cx. torrentium* are difficult to distinguish reliably based on morphological characteristics of adult females and were therefore grouped together as *Cx. pipiens/torrentium* [35].

### 2.2 Classification of environmental variables

Based upon the dispersal-range of *Cx. pipiens* s.l., the area within a 1500 m buffer around each trapping site was extracted for each land use class (LGN2020, WUR 2020) and soil type (Grondsoortenkaart van Nederland 2006, WUR-Altera 2006) using QGIS (version 3.16, Hannover; Development Team, 2022) [37]. For the 48 classes of the LGN2020 land use database (table S1) we calculated the Bray-Curtis dissimilarity distances for each location and performed a hierarchical cluster analysis, to reduce the number of classes, whilst preserving the differences [38,39] (fig. S2). For land use, the analysis generated four classes: urban, peri-urban, rural low and rural high. To determine the predominant soil-type at each location, we first relabelled the ten existing classes to three classes based upon the geological time period when it was formed (in the Netherlands), to capture the large-scale difference in soil hydrology and flora and fauna in the Netherlands (high and low): (1) artificial (Anthropocene), encompassing artificial areas (buildings), (2) peat/clay (Holocene), encompassing swampy grounds, heavy clay, light clay and peat and (3) sandy (Pleistocene), encompassing sand, loam, light loam and heavy loam (table S2) [31]. We excluded the water class, which contains large surface water bodies, such as rivers, lakes, and the sea, as none are suitable habitats for *Cx. pipiens/torrentium* [2]. The soil-type map was not up to date for artificial soil-types, due to urban expansion between 2006 and 2020. To correct for this, we relabelled all built-up area land use classes from the LGN2020 land use database to artificial soils. We calculated the Bray-Curtis dissimilarity distances for each location and performed a hierarchical cluster analysis, to group each location by the predominant soil-type (fig. S3). Not all combinations between soil-type and land-use were present in our sampling design. We therefore decided to combine land use and soil type into six mixed soil/land use classes assigned to the corresponding sampling site(s): peat/clay rural low (ADV), sandy rural high (ALM, ASN, OVD), sandy rural low (BGM, ZWM), artificial urban (LYS, RMP, UVW), sandy peri-urban (MST, NBD, RSL, WAC), and peat/clay peri-urban (UEK). These six soil/land use classes, represented by 14 locations, do not capture all soil/land use combinations in the Netherlands.

Climate data was taken from the KNMI Data Platform (Royal Netherlands Meteorological Institute) for each location, trapping week and for twelve time lags, to capture the cumulative legacy effects [40]. Each time lag corresponds to a period of one week beginning on Saturday (beginning of mosquito trapping) and ending on Friday (i.e. one week previous, two weeks previous, et cetera). As mosquito abundance is strongly associated with minimum temperatures and precipitation, we limited the climatic analysis to the minimum weekly temperature (minimum C°/week) and precipitation (total mm/week) [23,41,42].

### 2.3 Data analysis

Mosquito community composition differences between soil types and land uses were analysed by calculating Bray-Curtis similarity distances based on the presence/absence of mosquito species over the entire two-year period [38,39]. To test if the *Cx. pipiens/torrentium* abundances differ between the six soil/land use classes, we performed a Kruskal-Wallis test followed by Dunn's multiple comparisons post hoc test on the weekly female count data [43].

To capture the legacy effects and variation of weekly minimum temperature and precipitation (fig. S4) across all time lags we performed a principal component analysis (fig. S5) [44]. Principal component (PC) 1 appeared strongly temperature dominated, positively correlated to first three lags, and negatively correlated with longer time lags. Precipitation was captured in PC2 and was negatively correlated for all time lags. We used the principal component scores to test the effects of minimum temperature (PC1) and precipitation (PC2) in interaction with soil type and land uses on the weekly female *Cx. pipiens/torrentium* abundances with a Generalized Linear Mixed Model (GLMM) using a Poisson distribution and log link function [45]. This was followed by a Tukey multiple comparisons post hoc test to compare the slopes of the different soil/land use classes [46]. The six soil/land use classes, minimum temperature (PC1) and precipitation (PC2) were included in the model as fixed effect variables, whereas sampling site and year of sampling were included as random effect variables. The dispersion parameter in our GLMM, which is the ratio of the observed variance to the expected variance, did not indicate overdispersion. Data analyses were conducted with RStudio (R version 4.1.0; R Core Team, 2021).

## 3 Results

**Fig. 2:**
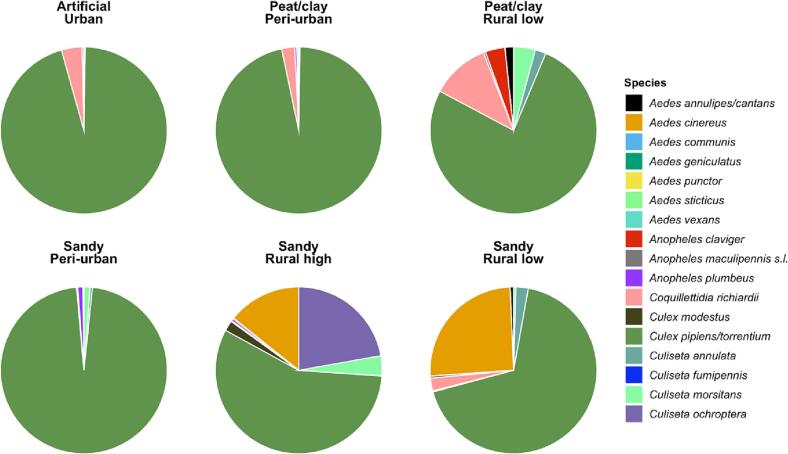
Mosquito species from 2020 to 2021 collected at the sampling sites spanning three soil types and four land use classes. In total, 9008 adult female mosquitoes of 17 species were trapped. For more information, see table S3.

In total, 9008 adult female mosquitoes from 17 species were trapped at the 14 sampling sites from 2020 to 2021 (Fig. 2, table S3). Four species/taxonomic groups, namely *Cx. pipiens/torrentium*, *Coquillettida richiardii*, *Culiseta annulata*, and *Anopheles maculipennis* s.l.*,* were present in all soil/land use classes. Most of the collected mosquitoes were *Cx. pipiens/torrentium* (80%). Mosquito species richness was highest on the non-artificial soils: sandy (14 species) and peat/clay (10 species) compared to artificial soils (4 species). Peri-urban land-uses (10 species) were more diverse than urban land uses (4 species) and were similar to rural land uses: rural low (9 species) and rural high (12 species). There were no trends distinguishable in the Bray-Curtis similarity distance (table S4). The overall community similarity within and between rural land uses across sandy and peat/clay soils is substantial, except between peat/clay rural low and sandy rural high. For further analyses we focused on *Cx. pipiens/torrentium* mosquitoes, as there were insufficient data for the other species.

### 3.1 The effect of soil and land-use on Cx. pipiens/torrentium abundances

**Fig. 3:**
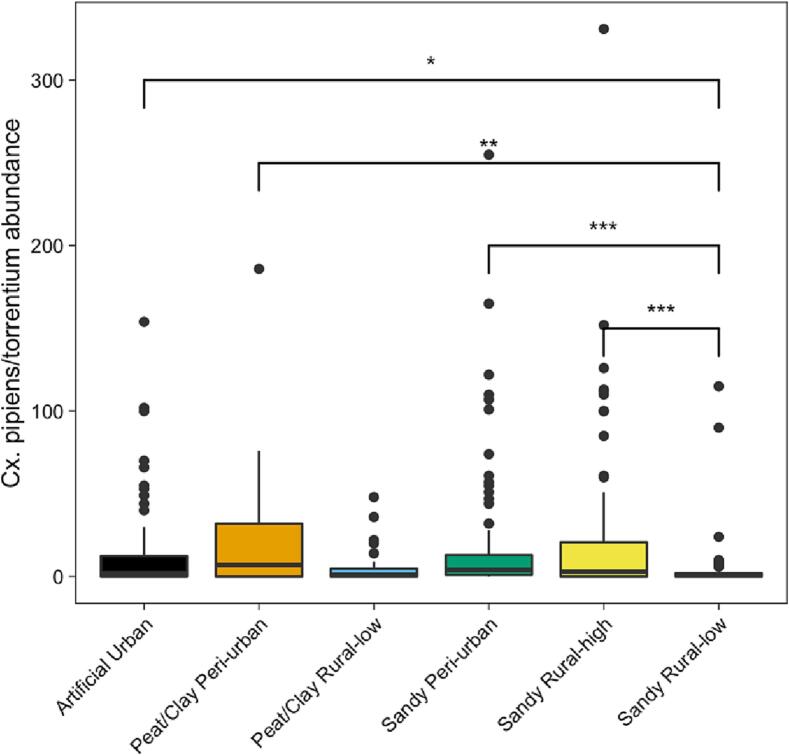
Mean number of female *Cx. pipiens/torrentium* mosquitoes captured at each soil type/land use class. Differences between groups are tested with a non-parametric Kruskal-Wallis' test and Dunn's multiple comparisons test. Significance codes: <0.001 ‘***’, <0.01 ‘**’, <0.05 ‘*’, >0.05 ‘ns.’.

Differences in *Cx. pipiens/torrentium* abundances were found across different soil/land use types. Overall, the highest *Cx. pipiens/torrentium* abundances were found in peat/clay peri-urban areas, whereas the lowest number of *Cx. pipiens/torrentium* were found in peat/clay rural low areas (Fig. 3). There was a statistically significant difference among soil/land use types, as assessed using the non-parametric Kruskal-Wallis test (x^2^ = 26.292, df = 5, *p* < 0.001). The pairwise Dunn's test between soil/land use classes showed that all soil/land uses except peat/clay rural low differed significantly from sandy rural low (Fig. 3, table S5).

### 3.2 Soil and land-use effects in interaction with minimum temperature and precipitation on Cx. pipiens/torrentium abundances

**Fig. 4:**
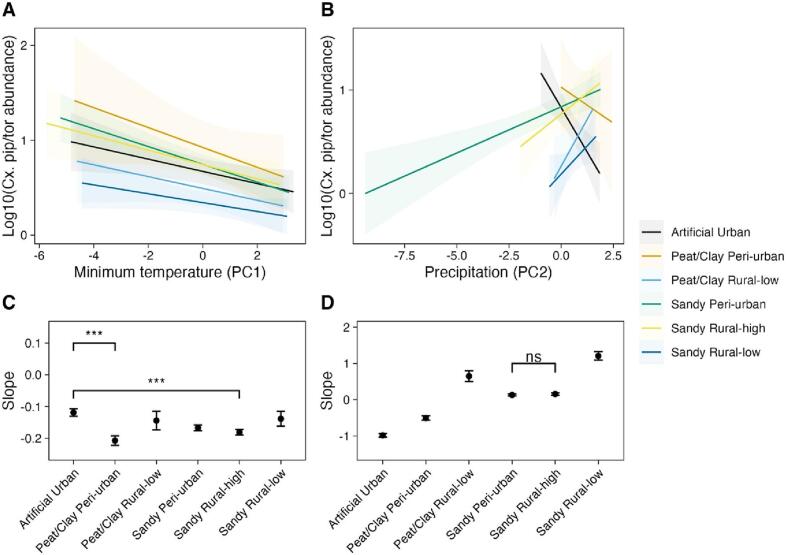
Relationship between soil type and land use on *Cx. pipiens/torrentium* abundances in response to minimum temperature (A-C) and precipitation (B—D) and as shown by the differences in slopes between soil/land use classes. All comparisons in panel D are significant except when indicated non-significant. Please note that the principal component values contain the variations of the twelve time lags. Significance codes: <0.001 ‘***’, <0.01 ‘**’, <0.05 ‘*’, >0.05 ‘ns.’.

Our results show that minimum temperature (PC1) had a significant interaction with most soil/land use classes in relation to *Cx. pipiens/torrentium* abundance. The abundance of *Cx. pipiens/torrentium* showed a negative relationship to PC1 (i.e. negative to short-term temperature and positive to long-term temperature) for all soil/land use types, with the exception for peat/clay rural-low and sandy rural-low (table S6). The slopes for *Cx. pipiens/torrentium* abundance in response to minimum temperatures differed significantly between artificial-urban vs. peat/clay-*peri* urban and artificial-urban vs. sandy-rural high (Fig. 4a, b, table S7). Also, the interaction between precipitation (PC2) and all soil land use classes was significant (table S8). All slopes were highly significant with *Cx. pipiens/torrentium* abundances being negatively correlated to PC2, i.e., positively to precipitation, in artificial urban and peat/clay *peri*-urban classes, whereas positive correlations were observed for the other classes (Fig. 4c, d, and table S9). This translated into significant differences in slopes for all pairs of soil/land use types with the exception of sandy-peri urban vs. sandy-rural high.

## 4 Discussion

The aim of this study was to investigate the independent and interactive effects of soil type, land use and climate parameters on the abundance of *Cx. pipiens/torrentium* mosquitoes, currently the most abundant and widespread vector species in Northwestern Europe. Interestingly, in our study, the overall highest abundance of *Cx. pipiens/torrentium* was found in the peat/clay peri-urban class. The site representing this class (UEK) was also the only location where WNV was observed in the Netherlands in 2020 [5]. However, although this association exists, the data is too scarce to draw any conclusions on the link between vector abundance and WNV transmission risk. There were a few instances where a soil type and land use class were represented by a limited number of locations: peat/clay rural low (represented by ADV) and peat/clay peri-urban (represented by UEK). One combination of soil type and land use, namely sandy rural low, only contained two locations: BGM and ZWM. Although the results from these categories may not be entirely generalizable due to the small sample size, these categories exhibit consistently different responses compared to the other classes.

Overall, our results show that most land use and soil types have a significant interaction between temperature, precipitation, and *Cx. pipiens/torrentium* abundances. While temperature effects were similar among land use and soil types, precipitation exhibited a markedly different impact on *Cx. pipiens/torrentium* abundances, especially in urban and peat/clay peri-urban soil/land use classes.

### 4.1 The interactive effect of soil/land use in response to precipitation

The observed precipitation-mediated differences in *Cx. pipiens/torrentium* abundances between soil/land use classes can partially be explained by *Cx. pipiens/torrentium* larval habitat preferences in relation to larval habitat availability. As stated earlier, *Cx. pipiens* s.l. and its sibling species can inhabit a wide range of larval habitats, including artificial habitats [2,35]. In general, land cover in urban areas consists mostly of artificial surfaces (i.e., pavements, buildings, gutters etc.), which are designed for high volume water drainage and are not well suited to deal with the small volumes of water that remain after rainfall and/or are dependent on maintenance to prevent blocking [47]. Additionally, backyards in urban and peri-urban residential areas are often full of potential breeding sites such as plant pots, rain barrels and other artificial containers, which fill up with water after rainfall or irrigation [26]. Such temporary water bodies are often devoid of predators and can therefore serve as long-term larval habitats without predation risk [26,29]. This may lead to very different dynamics in abundance of larval habitats compared to areas with a predominance of natural larval habitat [33]. Indeed, locations in both these land use classes are the ones that react very differently to precipitation. However, this appears to be only the case for urban and peri-urban land uses on artificial and peat/clay soils and not for peri-urban land uses on sandy soils. This might indicate that both the degree of urbanization and the presence of impermeable surfaces (artificial and clay) play an important role in why urban and peri-urban land uses on artificial and peat/clay soils react differently to precipitation than peri-urban land uses on sandy soils. This may operate as follows: after rainfall, water will remain present on the impenetrable surfaces in these urban and peri-urban locations for a prolonged period, subsequently leading to the availability of suitable habitats for the aquatic life stages of mosquitoes [33]. In contrast, sandy soils are generally more permeable than artificial and peat/clay, which leads to adequate water infiltration [31]. In rural land uses, we observe a different dynamic; in peat/clay areas the overall number and variation in the abundances of *Cx. pipiens/torrentium* is low compared to sandy rural areas. This might be due to the lower permeability of peat and clay soils, with clay acting as a barrier on which water can remain and peat acting as a sponge, absorbing excess water via swelling and releasing water as it shrinks [31]. Peat and clay soils are thus less dependent on precipitation in maintaining permanent water bodies. These permanent water bodies often have an established vertebrate- and macroinvertebrate community, which includes potential predators of mosquito larvae [33,48,49]. Sandy soils are more dependent on precipitation for maintaining permanent water bodies, resulting in a decline of permanent water bodies during droughts, and consequently an increase in the number of temporary water bodies after rain.

### 4.2 The interactive effect of soil/land use in response to minimum temperature

We observed a strong relation between temperature and *Cx. pipiens/torrentium* abundance, regardless of most soil/land use type. However, the relationship between minimum temperature and land use type was not statistically significant for the rural-low land use in both peat/clay and sandy soils. The sampling sites clustered within these soil/land use classes were situated near a large water body, which might play a role in the effects of temperature on *Cx. pipiens/torrentium* abundances. Overall, we observe that slope directions were similar irrespective of soil/land use type. It is possible that the observed lack of effect of temperature is a result of the sampling design, which involved placing mosquito traps at bird ringing stations located in areas with high woody vegetation. Vegetation can modify microclimatic conditions, including temperature, resulting in a similar environment for mosquitoes across different soil/land use types in terms of minimum temperature. Temperature plays an important role in the physiology, development and survival of mosquitoes and is a strong determining factor for the seasonality in mosquito abundances [33,35]. To a large extent, this is explained by the effect of temperature on mosquito larval development time, with an increase of temperature drastically shortening development time [33,50]. Subsequently, the decrease in development time may lead to an increase of mosquito abundance. As we sampled during the entire summer and during early autumn, our results likely reflect this phenomenon: higher temperatures lead to higher mosquito abundances regardless of soil type. As the autumn proceeds, temperatures drop across all locations and mosquito abundances decrease concomitantly.

## 5 Conclusion

Our results imply that precipitation affects *Cx. pipiens/torrentium* abundances differently across land use- and soil types. Our findings indicate that the interaction between precipitation and *Cx. pipiens/torrentium* abundances is influenced by soil type but can be overridden by (peri-)urban land uses, which can alter the availability of artificial breeding habitats. Conversely, the relationship between minimum temperature and *Cx. pipiens/torrentium* abundances appears to be consistent across land use and soil types. However, our sampling design may have contributed to this result, as our mosquito traps were located in areas with high woody vegetation. This could suggest that patches of high woody vegetation may serve as suitable habitats for adult mosquitoes, irrespective of land use. These results highlight the value of incorporating soil type and land use in conjunction with climatic variables in models to accurately estimate mosquito abundances at higher spatial and temporal resolution than would be possible with climatic variables alone. Overall, our study provides a proof of principle for the importance of soil type and land use for mosquito abundances. As mosquito species differ profoundly in their ecological preferences, particularly in the larval stages, these results cannot be uncritically extrapolated to other mosquito species of medical- and veterinary importance, such as invasive *Aedes*-species [33,35]. However, it can be hypothesized that species that require similar larval habitats to *Cx. pipiens*, like *Cs. annulata*, may react very similarly to differences in land use and soil type. These insights might help to inform management strategies and interventions to mitigate disease risk.

## Funding

This publication is part of the project ‘Preparing for vector-borne virus outbreaks in a changing world: a One Health Approach’ (NWA.1160.1S.210) which is (partly) financed by the Dutch Research Council (NWO).

## Accessibility

All figures are made colour-blind inclusive, using the colour-blindness simulation tool of Dr. David Nichols, which can be accessed via https://davidmathlogic.com/colorblind.

## CRediT authorship contribution statement

**Louie Krol:** Conceptualization, Investigation, Data curation, Formal analysis, Visualization, Writing – original draft. **Rody Blom:** Conceptualization, Investigation, Writing – original draft. **Martha Dellar:** Formal analysis, Writing – review & editing. **Jordy G. van der Beek:** Validation, Writing – review & editing. **Arjan C.J. Stroo:** Resources, Writing – review & editing. **Peter M. van Bodegom:** Supervision, Writing – review & editing. **Gertjan W. Geerling:** Writing – review & editing. **Constantianus J.M. Koenraadt:** Conceptualization, Funding acquisition, Supervision, Writing - review & editing. **Maarten Schrama:** Conceptualization, Writing – review & editing, Supervision, Funding acquisition.

## Declaration of Competing Interest

The authors declare that the research was conducted in the absence of any commercial or financial relationships that could be construed as a potential conflict of interest.

## Data Availability

All mosquito trapping data is publicly available and can be found in the supplementary data as an csv-file.

## Appendix A Supplementary data

Supplementary data Image 1

Mosquito trapping datafile Image 2
