# Supplementary material for: Interactive effects of climate, land use and soil type on Culex pipiens/torrentium abundance
Source: One Health. 2023 Jun 21;17:100589. doi: 10.1016/j.onehlt.2023.100589 (PMC10320611; doi:10.1016/j.onehlt.2023.100589)
Supplement: Supplementary file 1 — Supplementary data [file mmc1.docx]

**Supplementary materials:** Interactive effects of climate, land use and soil type on *Culex pipiens/torrentium* abundance

**
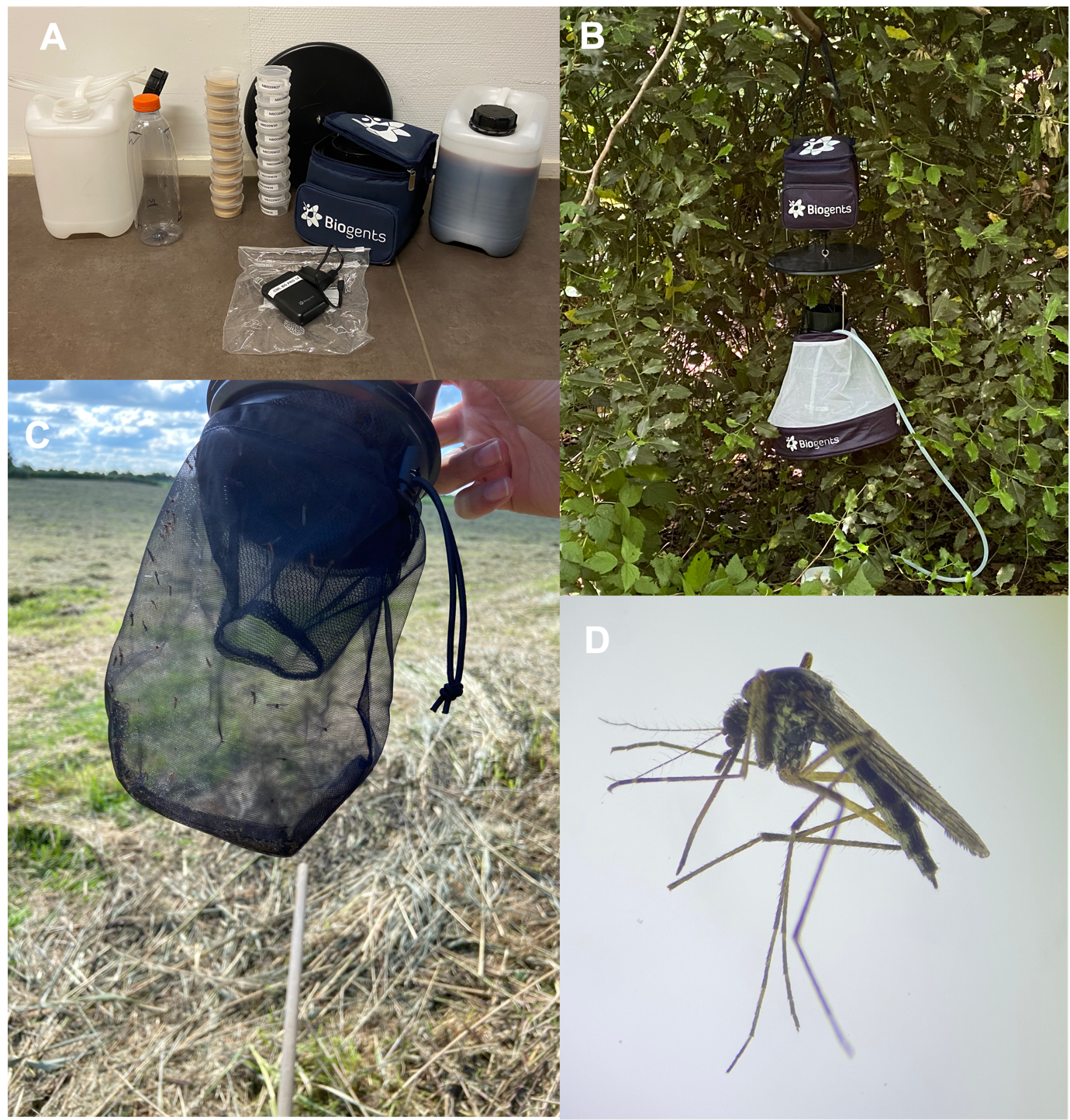
**

**Figure S1:** From early July to mid-October in 2020 and 2021, volunteers trapped mosquitoes at each sampling site on a weekly basis. Prior to collecting, each location was provided with mosquito trapping kits (A) and training in mosquito trapping. Together with the volunteers, we identified suitable locations to place the traps, typically at a height of 1-1.5 meters above ground level (B). After a single trapping night, volunteers emptied the traps (C) and stored the contents in a freezer at -20°C until we retrieved them. The female mosquitoes were then identified morphologically (D).


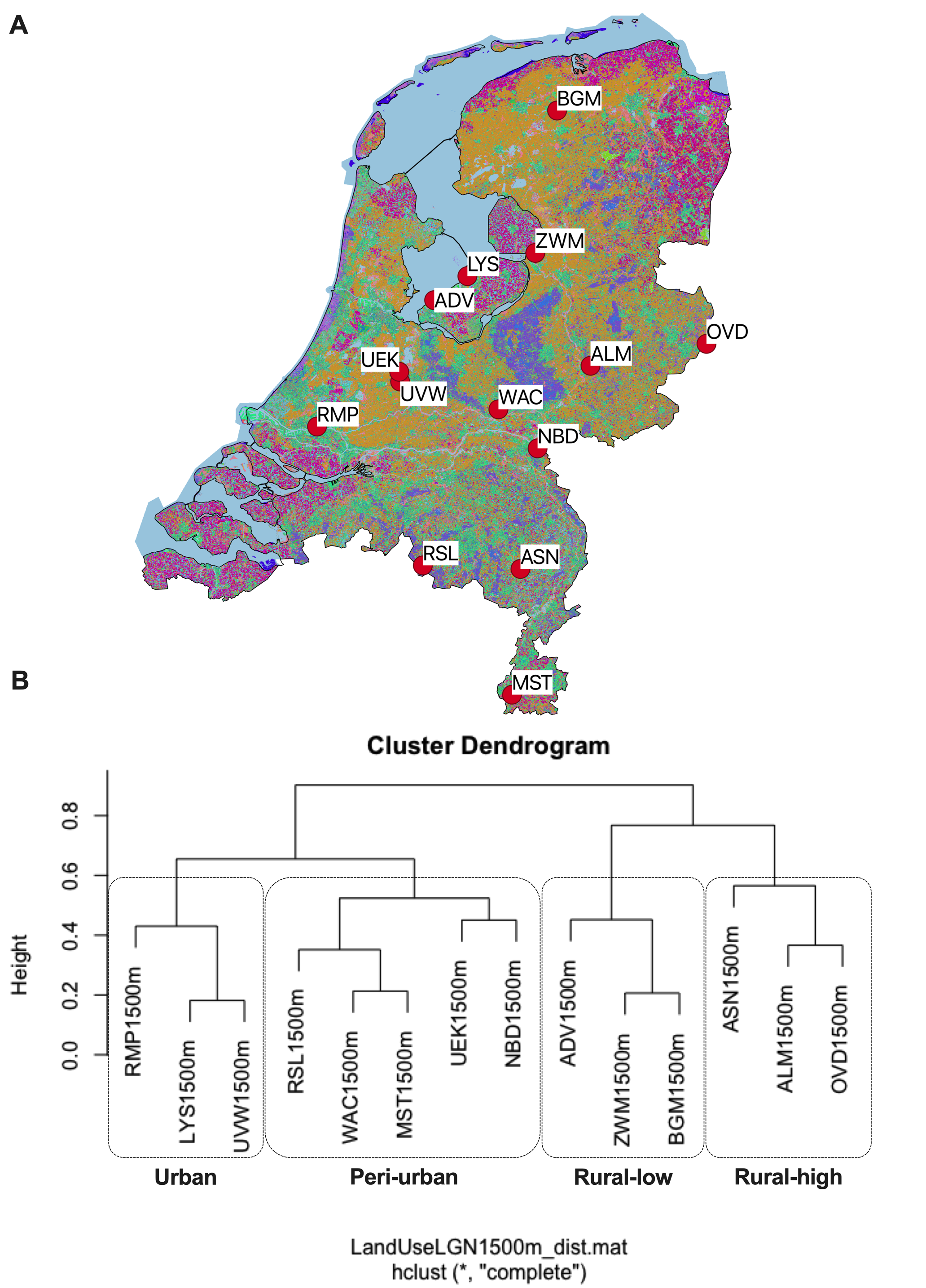


**Figure S2:** Mosquito sampling locations in the Netherlands in relation to the 48 land use classes (A). Data: WUR 2020: dataset LGN2020. Cluster dendrogram of land use classes (B), showing the four classes: urban, peri-urban, rural-high, and rural-low. See, table S1 for all land use classes from LGN2020.


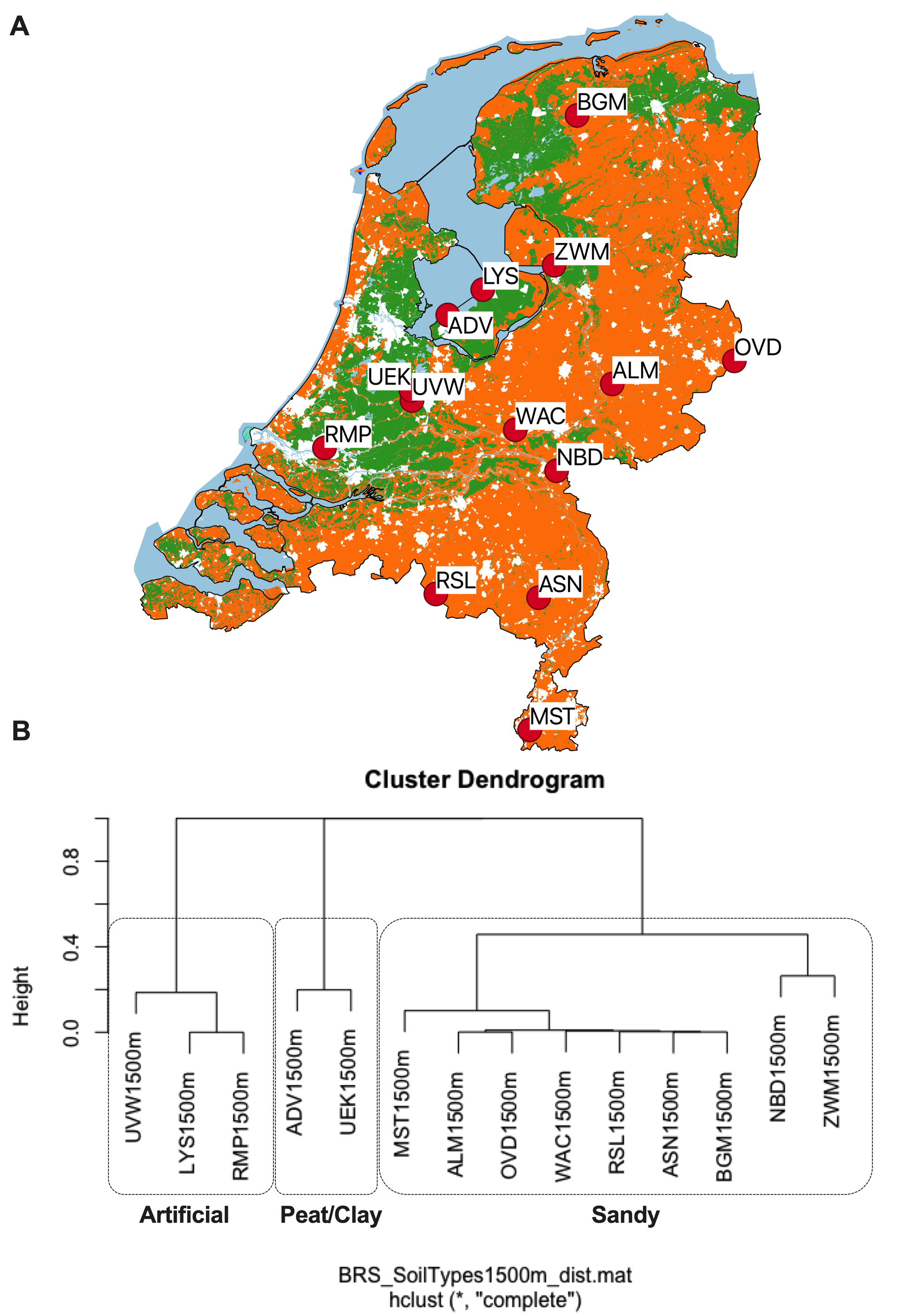


**Figure S3:** Mosquito sampling locations in relation to soil types (A), displaying sandy (orange), peat/clay (green), artificial (white) and water (blue). Data: WUR-Alterra, Grondsoortenkaart van Nederland 2006. Cluster dendrogram of sampling locations for soil type (B), showing the three classes: artificial, peat/clay and sandy. See, table S2 for all soil type classes.


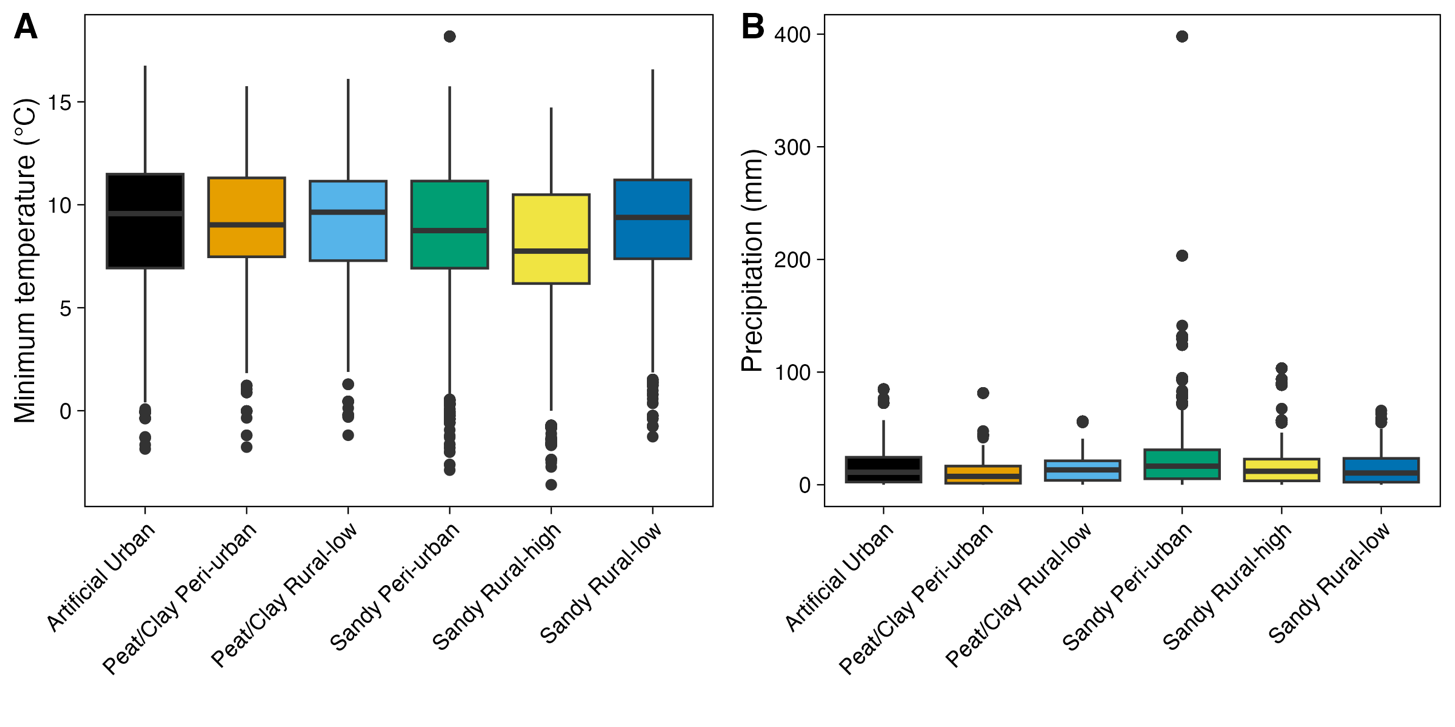
**Figure S4:** Ranges for the different temperatures (A) and precipitation (B) per the soil types and land uses.

**
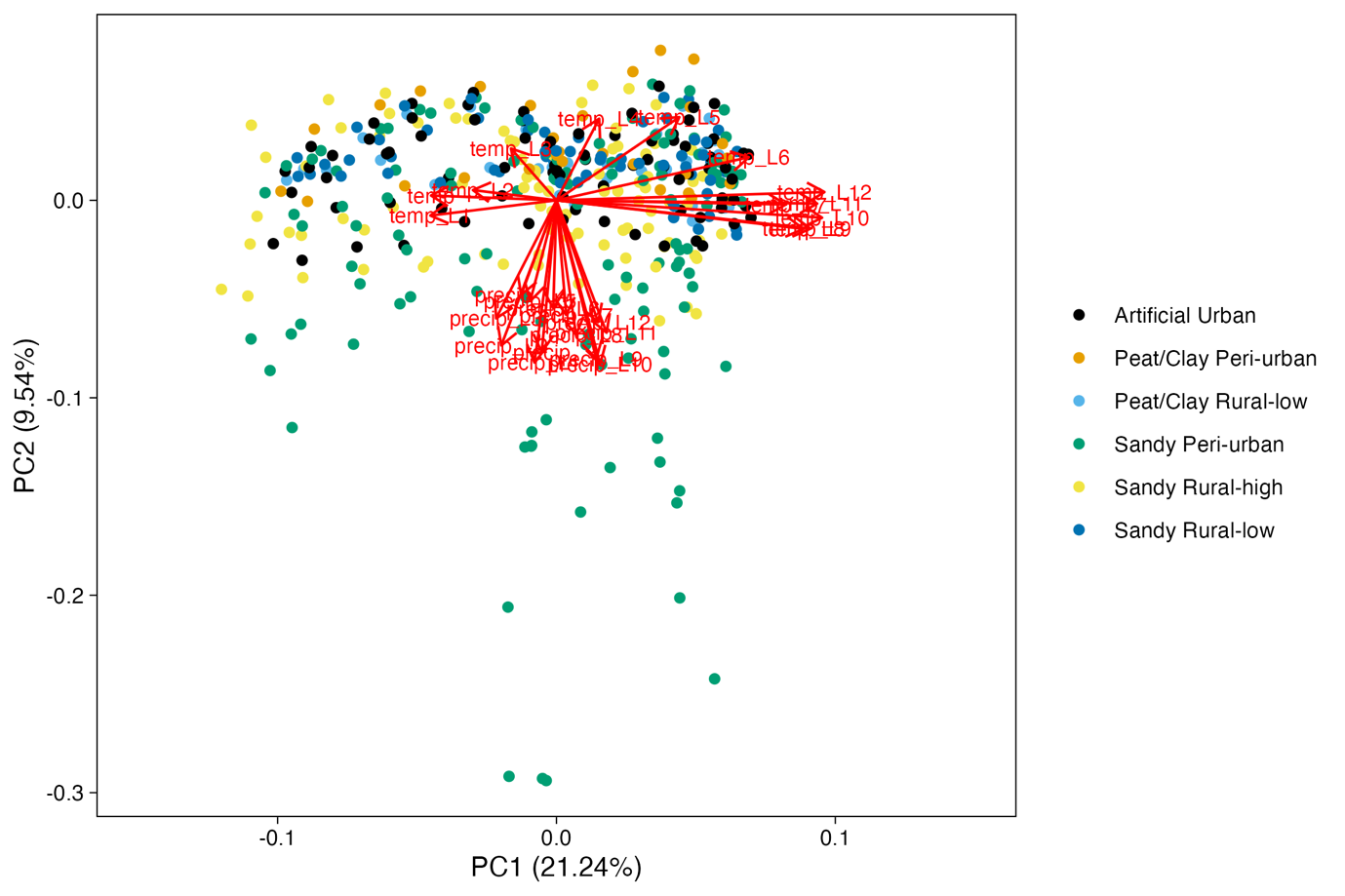
**

**Figure S5:** Principal component analysis (PCA) per time lag. Principal component (PC) 1 is strongly temperature dominated, positively correlated to first three lags, and negatively correlated with longer time lags. Precipitation was captured in PC2 and was negatively correlated for all time lags.

**Table S1:** Land use classes from LGN2020.

| Group | Description |
| --- | --- |
| Agriculture | Pasture |
|  | Maize |
|  | Potatoes |
|  | Sugar Beet |
|  | Cereals |
|  | Other agricultural crops |
|  | Greenhouses |
|  | Orchards |
|  | Flower bulbs |
|  | Tree nurseries |
|  | Fruit cultivations |
|  | Built-up areas outside urban areas |
|  | Other land use outside urban areas |
| Built-up area | Urban built-up areas |
|  | Semi-urban built-up areas |
|  | Bare soil built-up areas |
|  | Grass in built-up areas |
|  | Grass in semi-built-up areas |
|  | Forest in bult-up areas |
|  | Forest in semi-bult-up areas |
| Infrastructure | Main roads and railways |
| Water | Fresh water |
|  | Salt water |
| Forest | Deciduous forest |
|  | Coniferous forest |
| Nature | Salt marches |
|  | Coastal sands |
|  | Coastal dune areas with low vegetation |
|  | Coastal dune areas with high vegetation |
|  | Heathland in coastal areas |
|  | Grass in coastal areas |
|  | Heathland |
|  | Grassy heathland |
|  | Verry grassy heathland |
|  | Peat bogs |
|  | Bush/shrub vegetation in peat bogs (low) |
|  | Bush/shrub vegetation in peat bogs (high) |
|  | Forrest in peat bogs |
|  | Other swamp vegetation |
|  | Reeds |
|  | Bush/shrub vegetation in swamp areas (low) |
|  | Bush/shrub vegetation in swamp areas (high) |
|  | Forrest in swamp areas |
|  | Drifting sands/river sandbanks |
|  | Natural grasslands |
|  | Other grass |
|  | Other bush/shrub vegetation (low) |
|  | Other bush/shrub vegetation (high) |

**Table S2:** Relabelled and original label together with the geological time period of formation in the Netherlands, of soil types in the Netherlands (WUR-Alterra, Grondsoortenkaart van Nederland 2006).

| Relabelled | Original label | Geological time period of formation |
| --- | --- | --- |
| Artificial | **A**rtificial | Anthropocene |
| Peat/Clay | Swampy grounds | Holocene |
|  | Heavy clay |  |
|  | Light clay |  |
|  | Peat |  |
| Sandy | Sand | Pleistocene |
|  | Loam |  |
|  | Light loam |  |
|  | Heavy loam |  |
| Excluded | Water | n/a |

**Table S3:** Sampled mosquito-population from 2020-2021 at the sampling sites, in total, 9,008 adult female mosquitoes of 17 species were trapped.

| Species | Artificial Urban | Peat/Clay Peri-urban | Sandy Peri-urban | Peat/Clay Rural-low | Sandy Rural-low | Sandy Rural-high |
| --- | --- | --- | --- | --- | --- | --- |
| *Aedes annulipes/cantans* | 0 | 0 | 0 | 4 | 3 | 0 |
| *Aedes cinereus* | 0 | 0 | 1 | 0 | 116 | 450 |
| *Aedes communis* | 0 | 0 | 0 | 0 | 0 | 1 |
| *Aedes geniculatus* | 0 | 0 | 1 | 0 | 0 | 0 |
| *Aedes punctor* | 0 | 0 | 0 | 0 | 0 | 2 |
| *Aedes sticticus* | 0 | 1 | 0 | 0 | 0 | 0 |
| *Aedes vexans* | 0 | 1 | 0 | 0 | 0 | 0 |
| *Anopheles claviger* | 0 | 0 | 0 | 9 | 0 | 0 |
| *Anopheles maculipennis* | 4 | 5 | 1 | 1 | 2 | 2 |
| *Anopheles plumbeus* | 0 | 7 | 10 | 0 | 0 | 26 |
| *Coquillettidia richiardii* | 46 | 46 | 4 | 27 | 11 | 7 |
| *Culex modestus* | 0 | 0 | 0 | 0 | 1 | 64 |
| *Culex pipiens/torrentium* | 1099 | 1752 | 1904 | 182 | 310 | 2014 |
| *Culiseta annulata* | 3 | 3 | 6 | 5 | 11 | 6 |
| *Culiseta fumipennis* | 0 | 0 | 0 | 0 | 0 | 1 |
| *Culiseta morsitans* | 0 | 1 | 22 | 10 | 2 | 124 |
| *Culiseta ochroptera* | 0 | 0 | 0 | 0 | 0 | 700 |
| Total number of mosquitoes | **1152** | **1816** | **1949** | **238** | **456** | **3397** |
| Species richness | **4** | **8** | **8** | **7** | **8** | **12** |

**Table S4:** Mosquito community similarity between the soil types and land uses, using the Bray-Curtis similarity instance.

| Contrast | Artificial Urban | Peat/Clay Peri-urban | Sandy Peri-urban | Peat/Clay Rural-low | Sandy Rural-low | Sandy Rural-high |
| --- | --- | --- | --- | --- | --- | --- |
| Artificial Urban | 1.00 | 0.67 | 0.67 | 0.73 | 0.67 | 0.50 |
| Peat/Clay Peri-urban | 0.67 | 1.00 | 0.75 | 0.67 | 0.63 | 0.60 |
| Sandy Peri-urban | 0.67 | 0.75 | 1.00 | 0.67 | 0.75 | 0.70 |
| Peat/Clay Rural-low | 0.73 | 0.67 | 0.67 | 1.00 | 0.80 | 0.53 |
| Sandy Rural-low | 0.67 | 0.63 | 0.75 | 0.80 | 1.00 | 0.70 |
| Sandy Rural-high | 0.50 | 0.60 | 0.70 | 0.53 | 0.70 | 1.00 |

**Table S5:** Pairwise Dunn’s test between soil/land use classes. Significance codes: <0.001 ‘***’, <0.01 ‘**’, <0.05 ‘*’, >0.05 ‘ns.’. Showing a strong difference between sandy rural low and all other soil/land use classes, except for peat/clay rural low.

| Group 1 | Group 2 | n1 | n2 | statistic | p | p.adj |
| --- | --- | --- | --- | --- | --- | --- |
| Artificial Urban | Peat/Clay Peri-urban | 83 | 29 | 1.36509963 | 1.72E-01 | 1.0000 ns |
| Artificial Urban | Peat/Clay Rural-low | 83 | 30 | -1.280608 | 2.00E-01 | 1.0000 ns |
| Artificial Urban | Sandy Peri-urban | 83 | 120 | 1.0226839 | 3.06E-01 | 1.0000 ns |
| Artificial Urban | Sandy Rural-high | 83 | 90 | 0.91874973 | 3.58E-01 | 1.0000 ns |
| Artificial Urban | Sandy Rural-low | 83 | 60 | -3.1627277 | 1.56E-03 | 0.0234 * |
| Peat/Clay Peri-urban | Peat/Clay Rural-low | 29 | 30 | -2.1783413 | 2.94E-02 | 0.4407 ns |
| Peat/Clay Peri-urban | Sandy Peri-urban | 29 | 120 | -0.7174919 | 4.73E-01 | 1.0000 ns |
| Peat/Clay Peri-urban | Sandy Rural-high | 29 | 90 | -0.7242595 | 4.69E-01 | 1.0000 ns |
| Peat/Clay Peri-urban | Sandy Rural-low | 29 | 60 | -3.6717195 | 2.41E-04 | 0.0036 ** |
| Peat/Clay Rural-low | Sandy Peri-urban | 30 | 120 | 2.05173903 | 4.02E-02 | 0.6029 ns |
| Peat/Clay Rural-low | Sandy Rural-high | 30 | 90 | 1.9572482 | 5.03E-02 | 0.7548 ns |
| Peat/Clay Rural-low | Sandy Rural-low | 30 | 60 | -1.1767603 | 2.39E-01 | 1.0000 ns |
| Sandy Peri-urban | Sandy Rural-high | 120 | 90 | -0.0443573 | 9.65E-01 | 1.0000 ns |
| Sandy Peri-urban | Sandy Rural-low | 120 | 60 | -4.312974 | 1.61E-05 | 0.0002 *** |
| Sandy Rural-high | Sandy Rural-low | 90 | 60 | -4.0545345 | 5.02E-05 | 0.0008 *** |

**Table S6:** Fixed effects coefficients of the slopes from GLMM PC1 minimum temperature between soil/land use classes. Significance codes: <0.001 ‘***’, <0.01 ‘**’, <0.05 ‘*’, >0.05 ‘ns.’. Showing strong interactions with PC1 for all soil/land use classes with the exception of peat/clay rural-low and sandy rural-low, indicating that the slopes for those with significant interactions are significantly different from 0.

| Fixed effect | Estimate | SE | z.ratio | p.value |
| --- | --- | --- | --- | --- |
| Artificial Urban | -0.11886 | 0.01201 | -9.9 | <.0001*** |
| Peat/Clay Peri-urban | -0.08833 | 0.01909 | -4.626 | <.0001*** |
| Peat/Clay Rural-low | -0.02537 | 0.03199 | -0.793 | 0.42777 ns |
| Sandy Peri-urban | -0.04825 | 0.01506 | -3.203 | 0.00136 ** |
| Sandy Rural-high | -0.06217 | 0.01498 | -4.151 | <.0001*** |
| Sandy Rural-low | -0.01953 | 0.02646 | -0.738 | 0.4606 ns |

**Table S7:** Multiple comparison of the slopes from GLMM PC1 minimum temperature between soil/land use classes, using a Tukey post-hoc test. Significance codes: <0.001 ‘***’, <0.01 ‘**’, <0.05 ‘*’, >0.05 ‘ns.’. Showing a strong difference between artificial urban and peat/clay peri-urban and sandy rural high.

| Contrast | Estimate | SE | z.ratio | p.value |
| --- | --- | --- | --- | --- |
| Artificial Urban - Peat/Clay Rural-low | 0.02536 | 0.032 | 0.793 | 0.9688 ns |
| Artificial Urban - Peat/Clay Peri-urban | 0.08833 | 0.0191 | 4.626 | 0.0001 *** |
| Artificial Urban - Sandy Peri-urban | 0.04825 | 0.0151 | 3.203 | 0.0171 ns |
| Artificial Urban - Sandy Rural-high | 0.06217 | 0.015 | 4.151 | 0.0005 *** |
| Artificial Urban - Sandy Rural-low | 0.01953 | 0.0265 | 0.738 | 0.9772 ns |
| Peat/Clay Rural-low - Peat/Clay Peri-urban | 0.06296 | 0.0332 | 1.899 | 0.4027 ns |
| Peat/Clay Rural-low - Sandy Peri-urban | 0.02289 | 0.031 | 0.738 | 0.9772 ns |
| Peat/Clay Rural-low - Sandy Rural-high | 0.0368 | 0.031 | 1.188 | 0.8426 ns |
| Peat/Clay Rural-low - Sandy Rural-low | -0.00583 | 0.0379 | -0.154 | 1.0000 ns |
| Peat/Clay Peri-urban - Sandy Peri-urban | -0.04007 | 0.0174 | -2.304 | 0.1922 ns |
| Peat/Clay Peri-urban - Sandy Rural-high | -0.02616 | 0.0173 | -1.511 | 0.6573 ns |
| Peat/Clay Peri-urban - Sandy Rural-low | -0.0688 | 0.0279 | -2.469 | 0.1334 ns |
| Sandy Peri-urban - Sandy Rural-high | 0.01392 | 0.0127 | 1.093 | 0.8844 ns |
| Sandy Peri-urban - Sandy Rural-low | -0.02872 | 0.0253 | -1.136 | 0.8663 ns |
| Sandy Rural-high - Sandy Rural-low | -0.04264 | 0.0252 | -1.69 | 0.5381 ns |

**Table S8:** Fixed effects coefficients of the slopes from GLMM PC2 precipitation between soil/land use classes. Significance codes: <0.001 ‘***’, <0.01 ‘**’, <0.05 ‘*’, >0.05 ‘ns.’. Showing strong interactions with PC2 for all soil/land use classes, indicating that the slopes for those with significant interactions are significantly different from 0.

| Fixed effect | Estimate | SE | z.ratio | p.value |
| --- | --- | --- | --- | --- |
| Artificial Urban | -0.98106 | 0.05067 | -19.36 | <.0001*** |
| Peat/Clay Peri-urban | 0.4772 | 0.07653 | 6.236 | <.0001*** |
| Peat/Clay Rural-low | 1.62971 | 0.1561 | 10.44 | <.0001*** |
| Sandy Peri-urban | 1.11315 | 0.05796 | 19.205 | <.0001*** |
| Sandy Rural-high | 1.13712 | 0.05746 | 19.789 | <.0001*** |
| Sandy Rural-low | 2.18854 | 0.12801 | 17.097 | <.0001*** |

**Table S9:** Multiple comparison of the slopes from GLMM PC2 precipitation between soil/land use classes, using a Tukey post-hoc test. Significance codes: <0.001 ‘***’, <0.01 ‘**’, <0.05 ‘*’, >0.05 ‘ns.’. There is a noticeable distinction between artificial soils and other soil/land use categories. Among peat/clay and sandy soils, significant differences can be observed between the various land use classes within the peat/clay soils, as well as from all other soil/land use categories. However, there is no marked distinction between sandy peri-urban and sandy rural-high areas.

| Contrast | Estimate | SE | z.ratio | p.value |
| --- | --- | --- | --- | --- |
| Artificial Urban - Peat/Clay Rural-low | -1.63 | 0.1561 | -10.44 | <.0001*** |
| Artificial Urban - Peat/Clay Peri-urban | -0.477 | 0.0765 | -6.236 | <.0001*** |
| Artificial Urban - Sandy Peri-urban | -1.113 | 0.058 | -19.205 | <.0001*** |
| Artificial Urban - Sandy Rural-high | -1.137 | 0.0575 | -19.789 | <.0001*** |
| Artificial Urban - Sandy Rural-low | -2.189 | 0.128 | -17.097 | <.0001*** |
| Peat/Clay Rural-low - Peat/Clay Peri-urban | 1.153 | 0.1592 | 7.238 | <.0001*** |
| Peat/Clay Rural-low - Sandy Peri-urban | 0.517 | 0.1513 | 3.415 | 0.0084 ** |
| Peat/Clay Rural-low - Sandy Rural-high | 0.493 | 0.151 | 3.262 | 0.0141 * |
| Peat/Clay Rural-low - Sandy Rural-low | -0.559 | 0.1894 | -2.95 | 0.0375 * |
| Peat/Clay Peri-urban - Sandy Peri-urban | -0.636 | 0.0664 | -9.572 | <.0001*** |
| Peat/Clay Peri-urban - Sandy Rural-high | -0.66 | 0.0655 | -10.079 | <.0001*** |
| Peat/Clay Peri-urban - Sandy Rural-low | -1.711 | 0.1317 | -12.995 | <.0001*** |
| Sandy Peri-urban - Sandy Rural-high | -0.024 | 0.0426 | -0.562 | 0.9934 ns |
| Sandy Peri-urban - Sandy Rural-low | -1.075 | 0.1223 | -8.793 | <.0001*** |
| Sandy Rural-high - Sandy Rural-low | -1.051 | 0.1217 | -8.639 | <.0001*** |
